# Enrichment and amplification of secreted α-synuclein from cellular model and saliva of patients with synucleinopathies

**DOI:** 10.52601/bpr.2025.250014

**Published:** 2026-06-30

**Authors:** Wuxian Xu, Mingyue Luan, Zhaoxia Wang, Jianwen Deng, Li Zhu

**Affiliations:** 1 Sino-Danish College, University of Chinese Academy of Sciences, Beijing 100049, China; 2 State Key Laboratory of Cognitive Science and Mental Health, Institute of Biophysics, Chinese Academy of Sciences, Beijing 100101, China; 3 Department of Neurology, Peking University First Hospital, Beijing 100034, China; 4 College of Life Sciences, University of Chinese Academy of Sciences, Beijing 100049, China

**Keywords:** α-Synuclein, Parkinson’s disease, Seeding activity, RT-QuIC, Saliva biomarkers, Synucleinopathies

## Abstract

The aggregation and propagation of pathological α-synuclein (α-Syn) are central to the pathogenesis of synucleinopathies, including Parkinson’s disease (PD), multiple system atrophy (MSA), and dementia with Lewy bodies (DLB). This study focuses on the enrichment and amplification of secreted α-Syn from cellular models and the saliva of PD patients, utilizing trichloroacetic acid (TCA) precipitation to retain the post-translational modifications (PTMs) and seeding properties of extracellular proteins. By applying TCA precipitation, we successfully concentrated α-Syn from PD patient saliva and cell culture media while preserving critical PTMs (*e*.*g*., truncation and phosphorylation) that are essential to amyloidogenicity. Subsequent Western blot (WB) and real-time quaking-induced conversion (RT-QuIC) assays revealed that TCA-enriched α-Syn retained robust prion-like seeding activity, enabling the cross-cellular propagation of pathologically misfolded species. This workflow establishes TCA precipitation as a unique tool for capturing pathologically modified α-Syn from biofluids while maintaining their native seeding capacity. By integrating enrichment and amplification strategies, our findings advance the utility of saliva-based biomarkers for synucleinopathies and provide a translational platform to evaluate therapeutics targeting α-Syn transmission.

## INTRODUCTION

Synucleinopathies represent a group of neurodegenerative diseases that include Parkinson’s disease (PD), multiple system atrophy (MSA), and dementia with Lewy bodies (DLB) (van der Perren *et al.* 2020). These diseases are characterized by the abnormal accumulation of α-synuclein (α-Syn), an intrinsically disordered protein predominantly localized in presynaptic neurons, within pathological inclusions (Calo *et al.* 2016). Emerging evidence suggests that extracellular α-Syn species, secreted via exosomes or non-vesicular pathways (Iraci *et al.* 2021), can function as "seeds" that propagate pathologically misfolded α-Syn across neural networks, ultimately contributing to the development of neurodegenerative diseases (Calabresi *et al.* 2023). Post-translational modifications (PTMs) such as truncation, phosphorylation, and ubiquitination have been reported to enhance α-Syn amyloidogenicity and seeding capacity (Ghosh *et al.* 2017). These modified forms of α-Syn are thus considered key drivers of synucleinopathy pathogenesis. Cellular and animal models have been developed to study α-Syn aggregation, propagation, and neurotoxicity, providing valuable tools for disease modeling and therapeutic evaluation. Additionally, biochemical and biophysical approaches, such as Thioflavin T (ThT) fluorescence kinetics, transmission electron microscopy (TEM), and atomic force microscopy (AFM), enable detailed characterization of α-Syn fibril structure and stability, improving our understanding of its pathological mechanisms (Long *et al.* 2022a; Long *et al.* 2022b). Despite significant advancement in understanding α-Syn transmission, a major challenge persists in detecting these pathologically modified species in biofluids. This difficulty arises due to their low abundance and the technical limitations of conventional enrichment methods.

Current strategies for concentrating extracellular α-Syn, such as ultracentrifugation and immunoprecipitation (Khalaf *et al.* 2014), often compromise the structural integrity or functional properties of secreted proteins. For instance, ultracentrifugation may co-pellet contaminants or disrupt labile PTMs, while antibody-dependent approaches are limited by epitope accessibility and conformational bias. Real-time quaking-induced conversion (RT-QuIC) assays are commonly used to detect the prion-like seeding activity of α-Syn aggregates in clinically accessible specimens such as cutaneous tissues, cerebrospinal fluid (CSF), and saliva for early disease detection (Concha-Marambio *et al.* 2023; Hong *et al.* 2010; Luan *et al.* 2022, 2024; Peng *et al.* 2023; Shaheen *et al.* 2020). However, the diagnostic sensitivity of these assays remains significantly constrained by the exceptionally low abundance of pathological α-Syn species in biological samples (Bargar *et al.* 2021; Calabresi *et al.* 2023; Groveman *et al.* 2018; Kuzkina *et al.* 2021; Srivastava *et al.* 2022). These disadvantages impede the detection and characterization of bioactive α-Syn species in clinical samples, thereby restricting the development of biomarkers and therapeutic strategies targeting transmissible α-Syn (Du *et al.* 2021; Shaheen *et al.* 2020).

To address these challenges, we optimized trichloroacetic acid (TCA) precipitation as a robust, cost-effective method to enrich secreted α-Syn from the saliva of PD patients and cell-conditioned media. Unlike conventional techniques, TCA precipitation can capture the native state of secreted α-Syn while preserving PTMs such as truncation and phosphorylation, which are critical for amyloid formation. By applying this approach, we successfully concentrated secreted α-Syn that retained its PTMs and potent prion-like seeding activity, as demonstrated by Western blot (WB) and RT-QuIC assays respectively, in cellular models and patients’ saliva. This workflow not only overcomes the limitations of existing enrichment methods but also bridges the gap between biomarker discovery and functional validation, providing a translational platform to evaluate therapeutic targets aimed at halting α-Syn transmission in synucleinopathies.

## OVERVIEW OF THE PROTOCOL

This protocol presents an integrated workflow to investigate pathologically relevant α-Syn secretion and its prion-like seeding activity in both cellular models and patient-derived biofluids (*e*.*g*., saliva). The workflow comprises four key steps: (1) TCA precipitation to enrich low-abundance, secreted α-Syn while preserving its PTMs and seeding competence; (2) WB analysis to validate α-Syn recovery and characterize PTM profiles (*e*.*g*., truncation and phosphorylation); (3) Purification of α-Syn monomers as standardized substrates for seeding amplification; and (4) RT-QuIC to quantify the prion-like seeding activity of TCA-enriched α-Syn.

By leveraging TCA precipitation—a denaturing acid-based method—the protocol achieves a high-efficiency concentration of extracellular α-Syn from complex matrices (*e*.*g*., cell-conditioned media or saliva) without disrupting its amyloidogenic PTMs. Subsequent WB confirms the retention of modified α-Syn species, while purified α-Syn monomers serve as substrates to amplify pathological seeds. Crucially, RT-QuIC enables ultrasensitive detection of α-Syn seeding activity, directly linking TCA-enriched species to their capacity to propagate cross-cellular amyloid aggregation in neuronal models.

This systematic approach bridges biochemical enrichment with functional validation, offering a translational tool to dissect α-Syn secretion dynamics, evaluate its role in synucleinopathy progression, and screen therapeutics targeting transmissible α-Syn. The protocol is optimized for adaptability across experimental and clinical settings, supporting both mechanistic studies of amyloid transmission and biomarker development in neurodegenerative diseases.

## APPLICATIONS AND ADVANTAGES OF THE PROTOCOL

This protocol enables sensitive, clinically translatable analysis of pathogenic α-Syn by integrating TCA precipitation, WB, purification of α-Syn monomers, and RT-QuIC. Its key advantages include preserving pathologically critical PTMs (*e*.*g*., truncation, phosphorylation) and prion-like seeding activity of α-Syn—features often lost in conventional methods such as ultracentrifugation or immunoprecipitation. TCA efficiently concentrates low-abundance α-Syn from saliva or cell media without disrupting amyloidogenic PTMs, making it uniquely suited for detecting early PD biomarkers in non-invasive biofluids. Subsequent RT-QuIC quantifies seeding competence, with standardized α-Syn monomers’ propagation, bridging biochemical recovery with functional validation.

The workflow’s modular design supports three major applications. (1) Mechanistic insights: Maps α-Syn secretion dynamics in cellular models and evaluates propagation pathways (*e*.*g*., peripheral-to-CNS transmission). (2) Early diagnosis: Detects seeding-competent α-Syn species in saliva, offering a non-invasive tool for diagnosing PD and related synucleinopathies. (3) Therapeutic development: Facilitates assessment of clinical treatment outcomes and provides timely feedback on drug efficacy through targeting transmissible α-Syn species.

## LIMITATIONS OF THE PROTOCOL

1 Sample constraints: TCA precipitation enriches α-Syn from simple biofluids (*e*.*g*., saliva, cell-conditioned media) with minimal preprocessing. However, lipid-rich samples (*e*.*g*., plasma) require additional steps (*e*.*g*., lipid extraction, dilution) to prevent contaminant co-precipitation, which may affect RT-QuIC specificity.

2 Technical demands: RT-QuIC requires precise shaking (600 r/min double orbital), temperature control (±0.5°C), evaporation prevention (seal plate with tape) and acceleration (add glass bead in each well). Inexperienced users may misinterpret background noise as a seeding activity, necessitating careful training and validation.

3 Physiological limitations: Purified α-Syn monomers lack the diversity of patient-derived α-Syn, including strain-specific PTMs and co-aggregates. This may oversimplify cross-cellular propagation studies, limiting the physiological relevance of the results.

4 Clinical translation gaps: While RT-QuIC can detect α-Syn seeds in PD saliva, larger sample sizes and longitudinal data are required to establish diagnostic reliability. This remains an unmet need in synucleinopathy research, highlighting the challenges in translating laboratory findings to clinical applications.

## PROCEDURE

### TCA protein precipitation [TIMING ~1.5 h]

#### 1 Sample preparation

(A) Collect cell-conditioned media or biofluids (*e*.*g*., saliva) in pre-chilled 1.5 mL microcentrifuge tubes.

(B) For cell-conditioned media, centrifuge samples at 1000*g* for 5 min at 4°C to remove cell debris. For biofluids (*e*.*g*., saliva), centrifuge samples at 12,000*g* for 20 min at 4°C to remove food debris. Transfer the clarified supernatant to a new microcentrifuge tube.

#### 2 Primary TCA precipitation

(A) Add ice-cold 100% TCA (2.2 g/mL in H_2_O, freshly prepared) to the supernatant at a 1:4 volume ratio (*e*.*g*., 250 μL 100% TCA per 1 mL sample).

(B) Vortex gently for 10 s and incubate on ice for 10 min to denature proteins.

#### 3 Secondary TCA precipitation

(A) Add ice-cold 10% TCA to the mixture at a 1:2 volume ratio (*e*.*g*., 500 μL 10% TCA per 1 mL sample).

(B) Invert tubes to mix and incubate on ice for 20 min to enhance protein aggregation.

#### 4 Pellet recovery

(A) Centrifuge at 17,000*g* for 30 min at 4°C. Discard the supernatant without disturbing the pellet.

(B) Add 500 μL ice-cold acetone to wash residual TCA. Vortex briefly and centrifuge at 17,000*g* for 10 min at 4°C. Discard the supernatant.

#### 5 Protein resuspension

(A) Air-dry the pellet in a vacuum evaporator for ⩽5 min (avoid over-drying to prevent insolubility).

(B) Resuspend the pellet in 30–60 μL 1× loading buffer for WB or PBS for further experiments.

**[KEY NOTES]** (1) Temperature control: Keep samples on ice during all TCA precipitation steps to minimize protease activity and protein degradation. (2) Biofluid optimization: For biofluid samples (*e*.*g*., serum), pre-clear with organic solvents or adjust TCA ratios empirically.

### Western blot validation [TIMING 6~8 h]

#### 6 Gel preparation and electrophoresis

(A) Cast a 12% Tris-tricine SDS-PAGE gel (stacking gel: 4%; separating gel: 12%) using 1.5-mm spacers.

(B) Load 20−25 μL of TCA-precipitated protein (resuspended in 1× loading buffer) per lane. Include a pre-stained molecular weight marker (*e*.*g*., 8−180 kDa range).

(C) Run electrophoresis at 90 V in stacking gel (~15 min) and 140 V in resolving gel until the dye front reaches the bottom (~45 min for α-Syn detection).

#### 7 Wet transfer to nitrocellulose (NC) membrane

(A) Assemble transfer "sandwich" in ice-cold transfer buffer (25 mmol/L Tris, 192 mmol/L glycine, 20% methanol)

(B) Assembly by: filter paper, gel, NC membrane (0.22 μm), filter paper.

(C) Transfer at 300 mA for 1 h in a 4°C cold environment.

#### 8 Blocking and antibody incubation

(A) Block the membrane in 5% non-fat milk/TBST (Tris-buffered saline + 0.1% Tween-20) for 1 h at room temperature on a rocker.

(B) Incubate the membrane with primary antibody (*e*.*g*., anti-α-Syn [PTG-10842-1-AP, 1:4000] in 0.5% milk/TBST) overnight at 4°C with gentle agitation.

(C) Wash three times with TBST (5−10 min per wash).

#### 9 Secondary antibody and detection

(A) Incubate the membrane with a secondary antibody (*e*.*g*., anti-rabbit IgG, 1:10,000) in TBST for 1 h at room temperature.

(B) Wash three times with TBST (5−10 min per wash).

(C) Apply chemiluminescent substrate (*e*.*g*., ECL Prime, 1:1 mix of reagents A/B) for 1 min. Capture signals using a chemiluminescence imaging system (*e*.*g*., Beijing Sai Zhi, MiniChemi) with 1−10 min exposures.

**[KEY NOTES]** (1) Membrane choice: Compared to PVDF membrane, 0.22 μm NC membrane is chosen for its low cost, no activation requirement, and low background noise, making it ideal for capturing low-molecular-weight fragments, such as α-Syn. (2) Signal optimization: For weak signals, extend primary antibody incubation to 48 h at 4°C.

### α-Syn monomer preparation [TIMING 3 d]

Purification of α-Syn follows the previous protocol (Huang *et al.* 2005) with slight modifications.

#### 10 Bacterial culture and protein expression induction

##### 10.1 Pre-culture

(A) Inoculate glycerol stock of *E. coli* BL21(DE3) harboring the plasmid pET-3a containing human α-Syn cDNA into 10 mL LB medium supplemented with 50 μg/mL ampicillin (final concentration).

(B) Incubate overnight at 37°C with shaking (200 r/min).

##### 10.2 Large-scale culture and induction

(A) Transfer 10 mL of pre-culture into 1 L fresh LB medium containing 50 μg/mL ampicillin.

(B) Grow at 37°C, 200 r/min, until the optical density at 600 nm (*OD*_600_) reaches 0.6−0.8.

(C) Induce protein expression by adding 200 μL of 1 mol/L isopropyl β-D-1-thiogalactopyranoside (IPTG) (final concentration: 0.2 mmol/L).

(D) Continue shaking incubation at 37°C, 200 r/min, for 3–4 h.

##### 10.3 Bacterial harvesting

(A) Centrifuge the culture at 5000*g* for 15 min at 4°C to pellet cells.

(B) Resuspend the cell pellet in 50 mL lysis buffer (see Step 11) for purification or store at −80°C until lysis.

#### 11 Bacterial lysis

##### 11.1 Lysis buffer composition

20 mmol/L Tris-HCl (pH 7.4), 2 mmol/L EDTA, and 100 mmol/L NaCl.

##### 11.2 Lysis procedure

(A) Thaw bacterial pellets on ice and resuspend them in 50 mL lysis buffer. Stir at 4°C until the suspension is homogeneous and no large bacterial clumps remain.

(B) Add 50 μL of 100× PMSF to the lysis buffer to inhibit protease activity.

(C) Lyse cells using a high-pressure homogenizer, performing 4–5 cycles to ensure complete disruption.

(D) Boil the lysate in a water bath at 100°C for 15 min to denature unwanted proteins.

(E) Centrifuge the lysate at 18,500 r/min (~39,000*g*) for 50 min at 4°C using a refrigerated centrifuge to pellet cellular debris.

(F) Collect the supernatant (soluble fraction) for further purification.

#### 12 Protein purification (anion exchange chromatography)

##### 12.1 Buffer preparation

(A) Equilibration/binding buffer (Buffer A): 20 mmol/L Tris-HCl (pH 7.4) and 2 mmol/L EDTA.

(B) Elution buffer (Buffer B): 20 mmol/L Tris-HCl (pH 7.4), 2 mmol/L EDTA, and 1 mol/L NaCl.

##### 12.2 Chromatography steps

(A) Column equilibration. (1) Connect a HiTrap Q HP column (5 mL) to an ACTA Prime Plus system. (2) Equilibrate the column with 5 column volumes (CV) of Buffer A at a flow rate of 5 mL/min.

(B) Sample loading. Load the clarified cell lysate onto the column at 1 mL/min through the superloop.

(C) Wash and elute with linear gradient. Wash the column with 5 CV of Buffer A and then elute with a 0–1 mol/L NaCl gradient in Buffer A. Collect 2 mL fractions during elution.

##### 12.3 SDS-PAGE fraction analysis

(A) Mix 10 μL purified protein with 10 μL 2× loading buffer and incubate at 95°C for 5 min.

(B) Load samples onto a 15% SDS-PAGE gel (stacking gel: 5%) and run at 100 V (stacking) and 200 V (separation).

(C) Stain with Coomassie Brilliant Blue R-250 and destain to visualize bands.

(D) Pool fractions containing α-Syn.

#### 13 Size exclusion chromatography (SEC) and buffer exchange

##### 13.1 SEC buffer

40 mmol/L phosphate buffer (pH 7.4) and 170 mmol/L NaCl.

##### 13.2 Column equilibration

(A) Connect a Sephacryl S-300 column (120 mL) to the ACTA Prime Plus system and equilibrate with 1 CV of equilibration buffer at a flow rate of 0.5 mL/min.

(B) Confirm baseline stability by monitoring absorbance at 280 nm (baseline fluctuation < 0.005 AU).

##### 13.3 Sample preparation

(A) Membrane filtration. Filter the supernatant through a 0.22-μm syringe filter to eliminate particulate contaminants.

(B) Volume adjustment (for a 120 mL column). If the sample volume exceeds 5 mL (>5% CV), pre-concentrate using a 3-kDa molecular weight cutoff (MWCO) centrifugal filter (4000*g*, 4°C).

##### 13.4 SEC separation

(A) Sample loading and elution. (1) Load 5 mL of prepared sample onto the column at a flow rate of 0.5 mL/min. (2) Perform isocratic elution with equilibration buffer while monitoring the UV absorbance at 280 nm.

(B) Fraction collection. Collect fractions corresponding to the monomeric α-Syn peak.

##### 13.5 Post-purification processing

(A) Quality assessment. Analyze fractions by SDS-PAGE (15% resolving gel, Coomassie Brilliant Blue staining) to confirm purity.

(B) Sample concentration. Pool high-purity fractions and concentrate to 2–4 mL using a 3-kDa MWCO centrifugal filter (4000*g*, 4°C).

##### 13.6 Storage

Aliquot the concentrated sample and store it at −80°C for long-term preservation.

**[KEY NOTES]** (1) Establish a retention time-molecular weight calibration curve using pre-calibrated standards (*e*.*g*., BSA, thyroglobulin, cytochrome C). (2) Rinse the FPLC system with ultrapure water after each use. For long-term storage, follow the manufacturer’s protocol for ethanol preservation. (3) Ensure proper balancing of centrifuge tubes during all steps. Use pre-chilled rotors for temperature-sensitive procedures.

#### 14 Protein quality control

##### 14.1 SDS-PAGE purity analysis

The SDS-PAGE purity analysis is the same as described in Step 13.

##### 14.2 Concentration determination via UV absorbance (A_280_)

(A) Blank preparation. Use SEC Buffer (40 mmol/L phosphate buffer, pH 7.4, 170 mmol/L NaCl) as the blank.

(B) Sample measurement. (1) Dilute purified α-Syn in a storage buffer to achieve an A_280_ value within the linear range (0.3–1.0). (2) Measure the absorbance at 280 nm using a UV-Vis spectrophotometer.

(C) Concentration calculation. Use the predicted extinction coefficient (*ε* = 0.377 mL/(mg·cm)) for α-Syn to calculate the concentration (mg/mL) of α-Syn:

Concentration (mg/mL) = (A_280_ × Dilution factor) / *ε*.

##### 14.3 Storage

(A) Aliquot purified α-Syn into small volumes (*e*.*g*., 50–100 μL) and store at −80°C.

(B) Avoid >3 freeze-thaw cycles to prevent aggregation.

**[KEY NOTES]** (1) Monomer validation. Confirm monomeric state via dynamic light scattering (DLS) or native PAGE. (2) This protocol ensures high-yield production of endotoxin-free, monomeric α-Syn suitable for functional assays (*e*.*g*., RT-QuIC) and structural studies.

### RT-QuIC seeding activity assay [TIMING 2~3 d]

RT-QuIC assay of α-Syn follows protocol referencing the previous experience of our lab, as stated in Luan’s paper (Luan *et al.* 2022).

#### 15 Plate preparation

(A) Load a black 96-well plate (*e*.*g*., Nunc™) with 3-mm glass beads (1 bead/well).

(B) Pre-chill plate on ice to minimize premature fibrillization.

#### 16 Seed preparation

(A) Resuspend TCA-precipitated samples in 10 mmol/L PBS (pH 7.4). Centrifuge at 20,000*g*, 10 min, 4°C to remove insoluble debris.

(B) Dilute clarified supernatant to 0.1–1.0 mg/mL α-Syn.

(C) Add 5 μL of the sample (or negative control) per well.

#### 17 Reaction mix preparation

(A) Prepare RT-QuIC master mix: 10 mmol/L PBS (pH 7.4), 500 mmol/L NaCl, 0.0015% SDS (*w*/*v*, sterile-filtered), 10 μmol/L ThT (freshly diluted in PBS, protected from light), and 0.5 or 1.0 mg/mL recombinant α-Syn monomer (pre-filtered through 100 kDa MWCO spin filters).

(B) Add 95 μL master mix to each well, and seal the plate with optical adhesive film.

#### 18 Instrument setup

(A) Pre-heat a plate reader (*e*.*g*., BioTek SYNERGY4) to 37°C.

(B) Set shaking parameters: 600 r/min, double orbital mode, 1-min shaking and 29-min rest cycles.

(C) Configure fluorescence detection: Ex/Em = 450/480 nm, gain optimized to 80%–90% saturation for positive controls.

(D) The plate was sealed with a Greiner EASY seal (Greiner Bio-one, Germany) and incubated into a SYNERGY4 (BioTek) plate reader.

#### 19 Kinetic monitoring

(A) Run reactions for 48 h or 72 h, and record ThT fluorescence every 30 min.

(B) Terminate runs when negative controls (*e*.*g*. PBS with RT-QuIC master mix) show fluorescence <10% of positive controls.

#### 20 ThT fluorescence normalization

(A) Background fluorescence from negative controls was subtracted from each well to account for baseline signal variations.

(B) All data were normalized to the maximal fluorescence intensity of each plate and shown as ThT fluorescence (%).

## ANTICIPATED RESULTS

This protocol combines TCA precipitation and RT-QuIC to robustly profile pathogenic α-Syn in biofluids. TCA enrichment efficiently recovers extracellular α-Syn from HEK293T cell-conditioned media, and saliva from PD patients and MSA patients while preserving its PTMs and seeding competence. Purified α-Syn monomers, validated for homogeneity, serve as aggregation-free substrates to quantify the prion-like seeding activity of modified α-Syn. RT-QuIC confirms the potent seeding capacity of TCA-isolated α-Syn, inducing rapid amyloid formation, with WB verifying PTM integrity. The workflow’s adaptability across cellular models and clinical samples enables mechanistic studies of α-Syn transmission, therapeutic screening, and biomarker development in synucleinopathies. By bridging molecular profiles to functional pathogenicity, it offers high-fidelity insights for neurodegenerative disease research.

**[?TROUBLESHOOTING]**

Troubleshooting advices for this protocol can be found in Tables 1–4.

**Table 1:** Troubleshooting guide for TCA protein precipitation issues

Problem	Potential causes	Solutions
Low protein recovery or incomplete precipitation	(1) Insufficient TCA concentration or incorrect ratio (2) Incomplete removal of debris (3) Over-drying the pellet, leading to insolubility	(1) Optimize TCA ratios (*e*.*g*., increase 100% TCA to 0.3:1 for lipid-rich samples) (2) Centrifuge at 15,000*g* for saliva and 1000*g* for cell media to remove debris (3) Air-dry pellet for ≤5 min and resuspend immediately
Protein degradation	(1) Prolonged processing time at room temperature (2) Protease activity in biofluids	(1) Keep samples on ice throughout the procedure (2) Add protease inhibitors (*e*.*g*., PMSF) before TCA precipitation
Pellet difficult to resuspend	(1) Over-drying during air-drying step (2) Insufficient buffer volume	(1) Air-dry for ≤5 min and resuspend immediately (2) Increase buffer volume (*e*.*g*., 60 μL PBS or loading buffer)

**Table 2:** Troubleshooting guide for Western blot issues

Problem	Potential causes	Solutions
Weak or no signal	(1) Inefficient transfer to nitrocellulose (2) Low antibody binding (3) Excessive washing	(1) Extend transfer time to 1.5 h, or increase methanol content to 25% in transfer buffer (2) Optimize primary antibody dilution (*e*.*g*., 1:2000) and incubate for 48 h at 4°C (3) Reduce TBST wash time to 3−4 min per wash
High background noise	(1) Incomplete blocking (2) Cross-contamination during washing	(1) Block with 5% BSA instead of milk for phospho-specific antibodies (2) Increase TBST washing frequency (*e*.*g*., 3 × 10 min) and use fresh buffers
Uneven band intensity	(1) Uneven gel loading (2) Poor membrane contact	(1) Load equal protein amounts (*e*.*g*., 20–25 μL per lane) (2) Ensure even pressure in transfer sandwich

**Table 3:** Troubleshooting guide for α-Syn purification issues

Problem	Potential causes	Solutions
Low purity after anion exchange chromatography	(1) Overloading column (2) Insufficient lysate clarification	(1) Load ≤10 mg total protein per run (2) Centrifuge lysate at 40,000*g* for 1 h before loading
Protein aggregation during size exclusion chromatography (SEC)	(1) High protein concentration (2) Improper buffer pH or contaminants	Dilute the sample to ≤2 mg/mL before SEC
Low yield of purified α-Syn	(1) Inefficient lysis (2) Loss of protein during purification steps	(1) Ensure complete bacterial lysis using 4–5 cycles of high-pressure homogenization (2) Pool fractions containing α-Syn to maximize yield

**Table 4:** Troubleshooting guide for RT-QuIC assay issues

Problem	Potential causes	Solutions
Delayed or no ThT fluorescence increase	(1) Insufficient seeding activity in TCA-enriched samples (2) Suboptimal SDS or ThT concentration	(1) Increase seed concentration to 2 μg/mL or extend reaction time to 72 h (2) Optimize SDS concentration (0.001%–0.003%) to balance fibril formation
High background fluorescence in negative controls	(1) Contamination from reagents or equipment (2) Premature fibrillization due to temperature fluctuations	(1) Use fresh, sterile-filtered buffers and UV-treated consumables (2) Pre-chill plates on ice and maintain 37°C during incubation
Inconsistent fibril formation	(1) Variability in recombinant α-Syn batch (2) Uneven plate shaking	(1) Use monomeric, endotoxin-free α-Syn validated by SEC (2) Ensure consistent 600 r/min shaking during RT-QuIC

### Cell line-specific validation of α-Syn secretion

**Figure 1:**
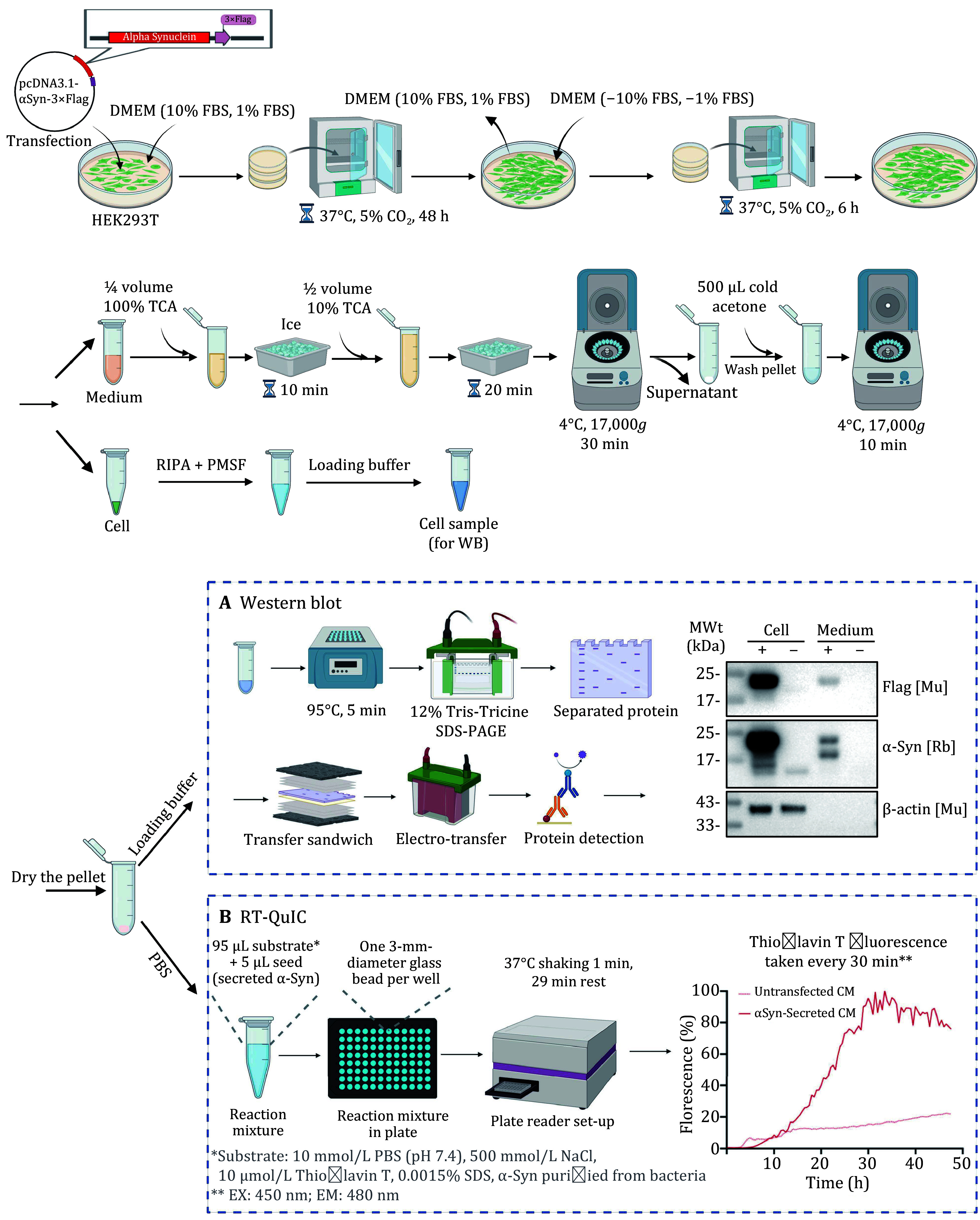
TCA precipitation efficiently captures extracellular α-Syn. HEK293T cells were transfected with pcDNA3.1-α-Syn-3×Flag, and α-Syn secretion was induced under serum-free conditions. TCA precipitation was used to concentrate extracellular α-Syn from a conditioned medium (CM), followed by WB analysis to confirm its presence (**A**). RT-QuIC assays demonstrated that TCA-precipitated α-Syn secreted from the cellular model retains seeding activity, indicating its potential role in prion-like aggregation (**B**)

TCA precipitation effectively captured extracellular α-Syn in both HEK293T and SH-SY5Y cell models. In HEK293T cultures, serum-free conditions contained only full-length α-Syn with a 3×Flag-tag (~23 kDa) detected by anti-Flag antibody (Fig. 1A, upper panel in WB image), and two bands (~23 kDa & ~17 kDa) detected by anti-α-Syn antibody (Fig. 1A, middle panel in WB image). The upper band should be full-length α-Syn with the 3×Flag-tag, while the lower band might be α-Syn without a Flag-tag compared to the band against anti-Flag antibody. Additional WB analyses were performed with a phosphorylation-specific α-Syn antibody, but no signal of phosphorylated α-Syn species was observed in the cell-conditioned media and even in the cell lysates (data not shown). HEK293T is a human kidney-derived embryonic cell, usually utilized as a cellular model in disease studies. Recent reports revealed that kidney dysfunction may contribute to α-Syn accumulation and its spread to the brain, implicating HEK293T cells as a relevant model for studying α-Syn clearance mechanisms and pathology (Yuan *et al.* 2025). Similarly, SH-SY5Y neuroblastoma cells which are one of the most widely used cellular models to study neurodegenerative diseases (Xicoy *et al.* 2017), secreted TCA-enriched full-length α-Syn with the 3×Flag-tag (~23 kDa) and truncations (~17–20 kDa) without a Flag-tag (data not shown). This indicates TCA treatment can maintain high recovery efficiency across both cell types, demonstrating robust applicability despite cell type-dependent truncation patterns.

### Patient saliva sample validation

**Figure 2:**
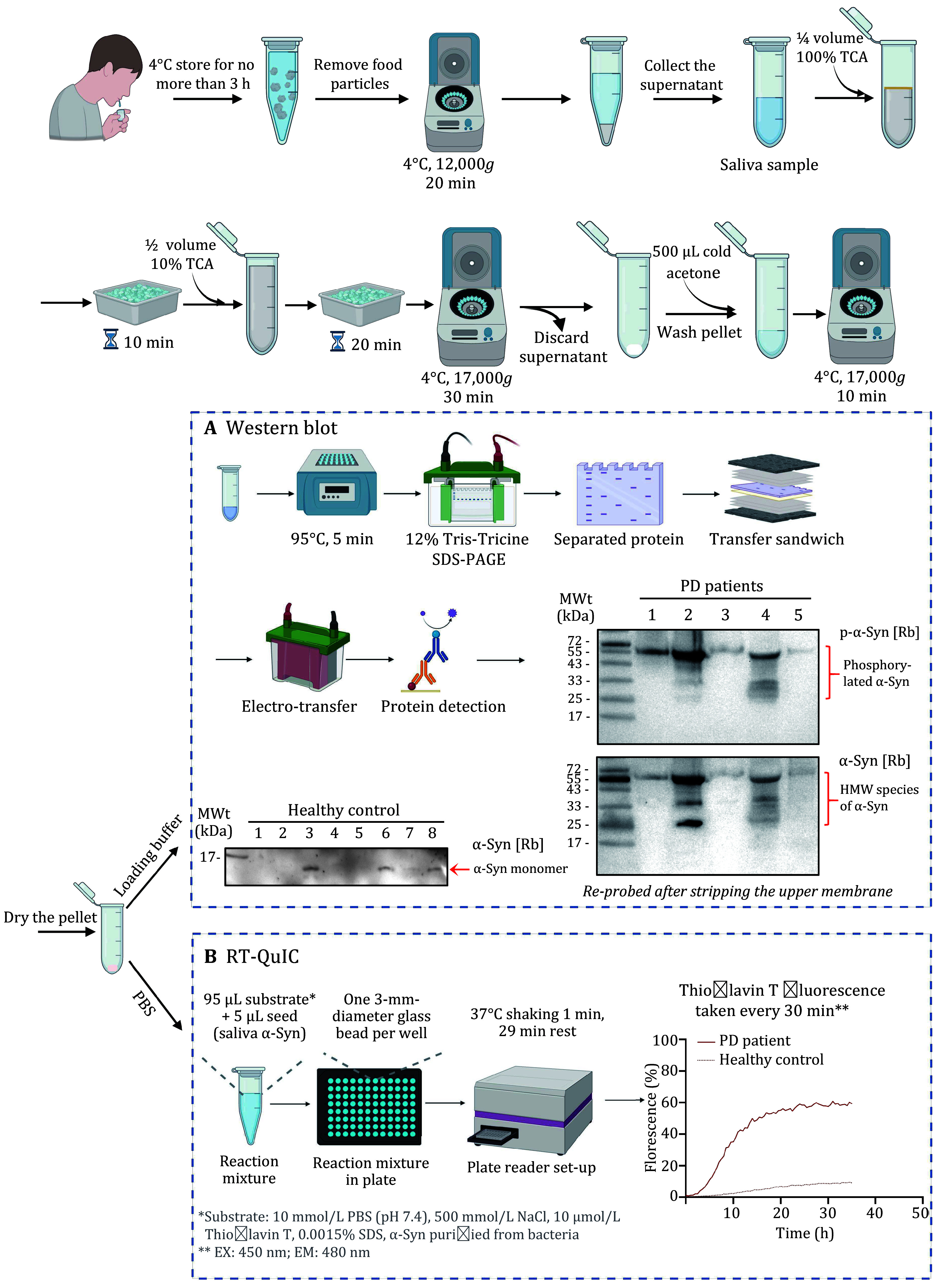
TCA precipitation enriches full-length and phosphorylated α-Syn in saliva samples from PD patients and healthy controls. Saliva samples were collected from PD patients and healthy controls, stored at 4°C for ≤3 h, and pre-cleared by centrifugation to remove food debris. TCA precipitation was performed to concentrate α-Syn, followed by WB analysis to detect full-length α-Syn in healthy control and phosphorylated α-Syn species in PD patients’ saliva samples. The lower left panel shows WB results from healthy control probed with α-Syn antibody, revealing prominent monomeric α-Syn bands at approximately 14 kDa. The upper right panel displays WB results from PD patient saliva samples probed with phospho-specific α-Syn antibody (p-α-Syn), indicating the presence of phosphorylated α-Syn species. The lower right panel shows the results of re-probing the same membrane with α-Syn antibody after stripping, revealing higher molecular weight (HMW) species of α-Syn (**A**). RT-QuIC assays demonstrated that TCA-precipitated α-Syn from PD patient saliva exhibits enhanced seeding activity, suggesting its potential as a biomarker for α-Syn aggregation (**B**)

TCA precipitation effectively enriched salivary proteins from both healthy controls and PD patients, as demonstrated by WB detection of α-Syn species. Healthy controls predominantly exhibited full-length α-Syn (~14 kDa, 3/9 cases, Fig. 2A, left WB image). In PD patients’ samples, a predominant band at ~55 kDa (5/5 cases), along with additional bands between ~33 kDa and ~25 kDa (2/5 cases), were detected using an anti-α-Syn antibody (Fig. 2A, lower right WB image), indicating the presence of higher molecular weight (HMG) species of α-Syn, possibly different forms of PTMs. Significantly, a strong signal was observed at ~55 kDa (3/5 cases) with an anti-p-α-Syn antibody, along with weaker signals detected at between ~33 kDa and ~25 kDa (2/5 cases) (Fig. 2A, upper right WB image), suggesting the presence of phosphorylated α-Syn species in PD saliva samples. Further analysis using silver staining combined with mass spectrometry (MS) is required to identify specific phosphorylation sites and other potential PTMs. The consistent presence of modified α-Syn in PD saliva but not in healthy control, aligned with full-length and truncated fragments from cellular models, confirms the capability of TCA precipitation to recover pathologically relevant species from clinical biofluids. Although larger sample sizes and longitudinal data are needed to establish diagnostic reliability, this methodological validation establishes TCA as a robust tool for salivary protein studies in PD biomarker discovery.

### Functional cross-validation by RT-QuIC

TCA-enriched α-Syn from both HEK293T cell-conditioned media and PD patients’ saliva exhibited potent seeding activity in RT-QuIC assays. Both sources triggered ThT fluorescence escalation within 18−24 h (Figs. 1B and 2B, RT-QuIC profile), with PD patients’ saliva achieving 12-fold higher maximum fluorescence than healthy controls (*p* < 0.001). The comparable lag phases (HEK293T: 12.3 ± 1.2 h; saliva: 10.2 ± 1.5 h) and amplification rates not only confirmed conserved pathogenic properties between cellular experimental and clinical α-Syn species but also implied stronger pathogenicity of phosphorylation modification.

## CONCLUSION

By demonstrating consistent α-Syn recovery and PTM retention in HEK293T cells, SH-SY5Y cells, and PD patients’ saliva, this protocol overcomes longstanding limitations of conventional enrichment methods. Its adaptability to diverse biofluids and cellular models, coupled with seamless integration into functional assays, positions TCA precipitation as an indispensable tool for synucleinopathy research. This protocol enables mechanistic studies of α-Syn transmission, biomarker discovery, and therapeutic screening with unprecedented fidelity.

## MATERIALS

### Reagents

• Trichloroacetic acid (TCA) (Sinopharm Chemical Reagent, Cat. No. 80243618)

**[CAUTION!]** TCA is corrosive. Wear gloves and eye protection. Neutralize waste with sodium bicarbonate before disposal.

• Acetone (Sinopharm Chemical Reagent, Cat. No. 10000492)

**[CAUTION!]** Acetone is flammable. Use in a well-ventilated area, away from open flames.

• 30% Acrylamide/Bis Solution (Beijing Lablead Biotech, Cat. No. A3291)

**[CAUTION!]** Acrylamide is neurotoxic. Wear gloves and a handle in a fume hood.

• TEMED (Ameresco, Cat. No. AAJ63734-AC)

**[CAUTION!]** TEMED is irritating to the skin and eyes. Use with PPE.

• Ammonium Persulfate (APS) (Beyotime, Cat. No. ST005)

**[CAUTION!]** APS is irritating to the skin and eyes. Use with PPE.

• Glycerol (Sinopharm Chemical Reagent, Cat. No. 10010618)

• Pre-stained Protein Marker (Yeasen Biotechnology, Cat. No. 20350ES72)

• Nitrocellulose Membrane (0.22 μm) (Filter technology, Cat. No.1215458)

• Tween-20 (Sinopharm Chemical Reagent, Cat. No. 30189328)

• Anti-α-Syn Antibody (Proteintech, Cat. No. 10842-1-AP)

• Anti-p-α-Syn Antibody (Abcam, Cat. No. ab317366)

• Anti-Rabbit IgG (Yeasen Biotechnology, Cat. No. 33101ES60)

• Super ECL Detection Reagent (Yeasen Biotechnology, Cat. No. 36208ES60)

• LB Medium (Beijing Lablead Biotech, Cat. No. LB0011)

• Ampicillin (Beijing Lablead Biotech, Cat. No. A9314)

• IPTG (Ameresco, Cat. No. 97061-776)

• EDTA (Sinopharm Chemical Reagent, Cat. No. 10009792)

• HiTrap Q HP Column (Cytiva, Cat. No.14115401)

• Coomassie Brilliant Blue R-250 (H&Z Life Science, Cat. No. 18.001.05)

• Thioflavin T (ThT) (Sigma Aldrich, Cat. No. T3516-5G)

**[CAUTION!]** ThT is harmful if inhaled. Handle with gloves and mask.

• Recombinant α-Syn Monomer (Prepared in-house; see Sections 12–14)

• Glass Beads (3 mm) (Fisher Scientific, Cat. No. 11-312A)

• DMEM medium (Gibco, supplemented with 10% FBS and 1% penicillin/streptomycin)

• Fetal bovine serum (FBS) (Gibco)

• Penicillin-streptomycin solution (Gibco, 100 U/mL)

• Trypsin-EDTA (Gibco, 0.25%)

• β-Mercaptoethanol (Sigma Aldrich, Cat. No. M6250-500ML)

**[CAUTION!]** β-Mercaptoethanol is volatile with a pungent odor. Make sure that wear a mask and operate in the fume hood during experiments. Dispose should be in accordance with local regulations.

### Reagents setup

• 1 mol/L anode buffer (10×) stock. Dissolve 121.14 g Tris in 900 mL deionized water and stir until it is dissolved. Adjust the pH to 8.9 with HCl. Bring the volume to 1 L and filter-sterilize the solution. This solution can be stored at room temperature.

• 1 mol/L cathode buffer (10×) stock. Dissolve 121.14 g Tris and 179.12 g Tricine in 900 mL deionized water and stir until it is dissolved. Adjust the pH to ~8.25 (do not correct the pH further). Bring the volume to 1 L and filter-sterilize the solution. This solution can be stored at room temperature.

• 3 mol/L gel buffer (3×) stock. Dissolve 363.42 g Tris in 900 mL deionized water and stir until it is dissolved. Adjust the pH to 8.45 with HCl. Bring the volume to 1 L and filter-sterilize the solution. This solution can be stored at room temperature.

• 12% separating gel stock. Dissolve the following components to prepare 9 mL of 12% separating gel for 2 × 0.75 mm gels: 30% A/B, 29:1, 3.3%: 3.6 mL, 50% Glycerol: 2 mL, 3× Gel Buffer: 3 mL, H_2_O: 0.4 mL, 10% AP: 45 μL, TEMED: 4.5 μL. Stir until fully dissolved. Pour the gel and allow it to polymerize. This gel can be used immediately for electrophoresis.

• 4% stacking gel stock. Dissolve the following components to prepare 5 mL of 4% stacking gel for 2 × 0.75 mm gels: 30% A/B, 29:1, 3.3%: 0.67 mL, 3× gel buffer: 1.24 mL, H_2_O: 3.09 mL, 10% AP: 40 μL, TEMED: 4 μL. Stir until fully dissolved. Pour the gel and allow it to polymerize. This gel can be used immediately for electrophoresis.

• TBST Stock. Prepare 1 L of TBST by adding 100 mL of 10× TBS and 1 mL of 0.1% (*v*/*v*) Tween-20 to 900 mL of deionized water. Mix thoroughly until the solution is fully homogeneous. This solution can be stored at room temperature.

• TBS Stock. Prepare 1 L of TBS by dissolving 80 g NaCl (1.37 mol/L, 2 g KCl (27 mmol/L), and 30 g Tris Base (248 mmol/L) in 800 mL of deionized water. Adjust the pH as desired (usually to pH 8) with 1 mol/L HCl. Bring the final volume to 1 L with deionized water and mix thoroughly until the solution is fully homogeneous. This solution can be stored at room temperature.

• Transfer buffer stock. Prepare 1 L of transfer buffer by adding 100 mL of anhydrous methanol and 100 mL of 10× transfer buffer to 800 mL of deionized water. Mix thoroughly until the solution is fully homogeneous. This solution can be used immediately for protein transfer.

• ThT stock solution. Dissolve 2 mg ThT in 1 mL PBS and mix thoroughly. Centrifuge at 13.3 r/min for 10 s at 4°C. Transfer the supernatant to a new tube. Dilute the solution 200-fold with 100% ethanol. Measure the OD416 using a spectrophotometer and calculate the concentration. Store the solution at 4°C.

• PMSF (0.2 mol/L). Dissolve 0.70 g PMSF in 16 mL of isopropanol. After ultrasonic-assisted dissolution, adjust the final volume to 20 mL. Aliquot into 1 mL portions, protect from light and store at −20°C. PMSF retains its stability under **[CAUTION!]** PMSF is acutely toxic. Wear a mask and handle with protective gloves.

• IPTG (1 mol/L). Dissolve 23.83 g IPTG in 90 mL of deionized water. Upon complete dissolution, adjust the final volume to 100 mL. Aliquot the solution and store at −20°C for long-term use.

• 500 mmol/L EDTA Stock. Dissolve 186.12 g EDTA in 900 mL of deionized water with continuous stirring until the majority of the solute is dissolved. Bring the final volume to 1 L and filter-sterilize the solution. The prepared solution is stable and can be stored at room temperature.

• Loading buffer (5×). Dissolve 5.0 g SDS in 8.33 mL of 1.5 mol/L Tris (pH 6.8). Next, carefully add 25 mL glycerol while avoiding the formation of air bubbles, and adjust the volume to 45 mL using deionized water. Once fully dissolved, incorporate 250 mg bromophenol blue and finalize the volume to 50 mL with β-mercaptoethanol. Ensure thorough mixing until the solution is completely uniform.

**[CAUTION!]** β-Mercaptoethanol is volatile with a pungent odor. Make sure that wear a mask and operate in the fume hood during experiments. Dispose should be in accordance with local regulations.

## EQUIPMENT

• Pipettes: 25, 10, 5, and 1 mL

• Centrifuge tube: 1.5, 2, 5, 15, and 50 mL

• 6-well plate (Biofil, Cat. No. TCP010006)

• pH meter (Sartorius, PB-10)

• Vortex (Qilinbeier)

• Pipettes (Eppendorf, 0.5−10, 2−20, 20−200, and 100−1000 μL)

• 96-well black microplate (Nunc)

• Cell culture incubator (Thermo Fisher Scientific, Series 8000)

• Inverted microscope (Nikon, TS100)

• Low-temperature high-speed centrifuge (Thermo Fisher Scientific, Fresco 17)

• Ultra-clean workbench (Suzhou Antai, BSC-1004IIA2)

• Biosafety cabinet (Suzhou Antai Air Technology Co., Ltd, BSC-1004IIA2)

• Shaking incubator (Shanghai Zhicheng, ZHWY-211C)

• ACTA Prime Plus system (Cytiva, 1456103)

• Ultrapure water system (Millipore, Milli-Q)

• SDS-PAGE electrophoresis apparatus (Bio-Rad, PowerPac HC)

• Western blot transfer system (Bio-Rad)

• Chemiluminescence imaging system (Beijing Sai Zhi, MiniChemi)

• Microplate reader (BioTek, Synergy 4)

• Thermostatic metal bath (Hangzhou Bori, CHB-100)

• UV spectrophotometer (Amersham Biosciences, Ultrospec 5300 pro)

• Negative pressure aspirator (Shanghai Baojia, YB-DX23D).

• −80°C freezer (Thermo Fisher Scientific)
